# Perceptions of the influence of a mobile phone-based messaging platform on caregiver ECD knowledge, attitudes and practices: a qualitative exploration in an informal settlement in Nairobi

**DOI:** 10.1186/s12875-023-02127-0

**Published:** 2023-09-14

**Authors:** Elizabeth Wambui, Margaret Nampijja, Kenneth Odhiambo Okelo, Ruth Muendo, Silas Onyango, Elizabeth W. Kimani-Murage, Patricia Kitsao-Wekulo

**Affiliations:** African Population and Health Research Center, APHRC Campus, Manga Close, Off Kirawa Road, P.O. Box 10787-00100, Nairobi, Kenya

**Keywords:** Early childhood development, Stimulation, Caregiver knowledge, Mobile phone technology

## Abstract

**Background:**

Programs supporting initiatives for children younger than three years are inadequate and not accessible to many families, particularly in resource-limited settings. Many primary caregivers have little knowledge on how to monitor the development of their children or the importance of engaging children in stimulative activities during the course of early development. Health system limitations make it difficult for health workers to educate and demonstrate stimulative engagement to caregivers. The massive use of technology can be used to facilitate access to growth and development programs for children. We developed and implemented a mobile phone technology to help caregivers monitor and stimulate their children’s development in real-time. This study explored the influence that this intervention had on the caregivers’ early child development (ECD) knowledge, attitudes and practices.

**Methods:**

In this qualitative cross-sectional study, we conducted interviews through eight (8) focus group discussions, three (3) key informant interviews and 9 indepth interviews among a total of 111 participants including primary caregivers (n = 87), community health volunteers (CHVs) (n = 21) health managers and workers (n = 3) to determine their attitudes and experience with the intervention with regards to improving their KAP. Interviews were audio-recorded, transcribed, and analyzed thematically.

**Results:**

Caregivers and CHVs reported that the intervention had provided them with new knowledge that positively influenced their ECD caregiving attitudes and practices. CHVs and health workers and managers reported that the intervention had provided caregivers with confidence in caring for their children while increasing their knowledge on how to monitor and stimulate their children’s development.

**Conclusion:**

Mobile phone technology can be effectively used to enhance caregivers’ knowledge of ECD and enable them to monitor and support their children’s development in real-time.

**Trial registration:**

The trial was registered with the Pan African Clinical Trial Registry (www.pactr.org) database (ID number: PACTR201905787868050 Date: 6/05/2019.

**Supplementary Information:**

The online version contains supplementary material available at 10.1186/s12875-023-02127-0.

## Background

The massive use of technology can be leveraged to provide access to growth and development programs for children. Programs supporting such initiatives for children younger than three years are rare and not accessible to most families, particularly in resource limited settings [1]. As a result, most primary caregivers are unable to identify delayed milestones in their children’s growth and development. Also, due to inadequate information, developmental delays are often reported late,when they have become severe and difficult to reverse [2].

Caregivers residing in low income communities are disadvantaged because of extreme poverty, food insecurity and low education levels [3]. These, together with other salient but important barriers including the lack of knowledge on ECD and inability to provide appropriate stimulation for young children [4], emanating from cultural and religious beliefs, single parenthood and limited father involvement negatively influence child development [5]. Residents in informal urban settlements tend to become parents at a much earlier age, with over half of mothers of children under five years aged below 25 years and about 25% being adolescents. These parents have low education levels, lack knowledge and therefore provide poor parenting practices that affect child nutrition and health [6].

It is essential to empower parents (primary caregivers) with the necessary information on ECD and enhance their skills in order for them to care for and stimulate their children for optimal growth and development. Infact, studies show that interventions targeting primary caregiver on nurturing care practices are effective at better developmental outcomes in their children [7]. For optimal development, children should be exposed to high quality and affordable ECD programs [8] that integrate ECD knowledge with an element of how caregivers can assess their children’s day-to-day activities to detect any developmental anormalies for immediate intervention [9]. Previous studies have looked at the delivery of health services by community health volunteers (CHVs) to caregivers using mobile phone based platforms. These studies note that the use of mobile-based modes of service delivery is largely welcome as it allows CHVs to work efficiently and make better connections with caregivers and community members at large [10]. In cases where caregivers have had acess to online phone-based applications, these applications are limited as they are not tailored to many caregivers’ contexts [11]. This however demonstrates the information gap that caregivers seek to fill and suggests that it is possible to utilize mobile based technology to bridge the gap in caregiving knowledge and practice thereby effecting better developmental outcomes for children. The use of mobile phones within the health sector in Kenya is progressively playing an important role in health care delivery and especially in tracking health outcomes.

Studies that leverage on mobile phone convenience show that this mode of engagement with caregivers is acceptable, cost-effective and provides better adherence to services [12]. Since children’s development takes place in daily life activities, caregivers should be able to note early signs of developmental delay during their regular interactions with their children. To our knowledge, no studies have examined the use of mobile phone platforms on caregiver knowledge attitude and practices. We therefore conducted a project to explore perceptions on the use of an innovative mobile phone platform to improve caregivers’ ECD knowledge attitudes and practices in a low income setting.

## Methods

### Study design

We conducted a phenomenological cross-sectional study among caregivers, CHVs and health workers involved in a mixed methods study, with participants recruited from Korogocho, Nairobi. The detailed methodology for the mixed methods study is provided elsewhere [13]. The mothers and CHVs were trained on how to record developmental milestones achieved by their children in real time, using a phone in order to help them identify and address developmental delays early by providing the necessary stimulation or medical help. The mothers were also given information of activities they could do with their children so as to enhance their development in various domains. The innovation relied on real-time data collection and feedback, coupled with home visits by community health volunteers (CHVs) who observed how caregivers were progressing with the intervention and provided support where necessary. Participants were interviewed after six months of implementing the messaging platform and again after twelve months of the same intervention.

### Study setting and participants

The study was conducted in Nairobi County among caregivers and children in Korogocho Ward within Ruaraka sub-County. Nairobi County is among the 47 semi-autonomous county governments which were formed after the promulgation of the current Kenyan constitution. Every county is further subdivided into sub-counties and wards for ease of administration. Nairobi County had a total population of 4,735,000 in 2019, with a population growth rate of 3.93%. The county has 17 administrative sub-counties of which Ruaraka is one.

Ruaraka sub-County has five wards (Baba Dogo, Utalii, Mathare North, Lucky Summer and Korogocho) with a total population of 192,620 (Baba Dogo 30,741, Utalii 36,275, Mathare North 53,658, Lucky Summer 30,000 and Korogocho 41,946). Our previous work in urban poor settings in Kenya indicates high levels of early motherhood, with women aged less than 20 years comprising close to 25% of mothers of children under five [14]. According to unpublished data from 2017, within Korogocho slum, almost 2000 mothers of children under five were aged 15–19 years and this made it an ideal setting for the project. Poor child feeding and care practices have been documented among these mothers [4], which result in poor child health outcomes, with close to 50% of children being stunted. The infant mortality rate is 57 deaths per 1000 live births [3]. Young mothers lack appropriate childcare knowledge and have limited ability to make childcare decisions and they typically rely on other older women for guidance [4]. While in general this guidance and support may be good, young mothers appear unable to challenge it. In the majority of these women, poverty-related challenges limit the opportunities for them to provide appropriate stimulation for their children. Struggles with motherhood and childcare, with little professional support often leaves the young mothers stressed, limiting their ability for childcare [4]. Thus, many children in urban poor settings maybe at risk of not achieving their developmental potential, therefore needing innovative interventions to improve parental support.

Primary caregivers and their children aged between 6 and 24 months and residing in the village were invited to participate and were provided with the intervention.

In each village, households with children in the eligible age-group were identified using the Nairobi Urban Health and Demographic Surveillance System (NUHDSS) database. The NUHDSS is conducted annually by the African Population and Health Research Center (APHRC) in Korogocho ward. A list of all potential participants was generated and the required number was randomly selected and approached about the study. We identified and recruited CHVs within the respective villages who in turn assisted the study team to locate the mothers/ caregivers who were eligible for inclusion into the study and to invite them to participate. A total of 135 caregivers were recruited into the intervention. Those assigned into the intervention had their children measured to verify their developmental age. This information together with their phone numbers was entered into a platform which was programmed to send questions to these caregivers to assess the psychomotor developmental progression of their children while providing care information to the caregivers on how to stimulate growth and developmental response for their children. In this study, caregivers in the intervention arm were purposively selected to participate in the focused group discussion and in-depth interviews. CHVs in the villages were used to inform and invite the participants to the various data collection forums. The interviews were held in a neighboring community school on a specified day, in a school hall that was provided.

### Data collection

We collected qualitative data at two time points that is, after six months of the intervention and after twelve months of the intervention. These interviews were conducted between between 27th January and 7th March 2020 and between 10th August and 21st September 2020 respectively. This study reports data collection from both time points.

At either data collection time points, the interviews were administered to caregivers, CHVs, healthcare managers and workers at the sub-county level as appropriate, to determine their perceptions on the intervention including its importance and how it had changed knowledge and care practices among caregivers. Interview guides for each interview type were prepared by the study team and piloted prior to the data collection exercise.

We conducted focus group discussions (FGDs) with CHVs and both female and male caregivers. CHVs were interviewed on their experience with the mobile application and their perceptions on the feasibility of its to track children’s developmental progression. We also conducted key informant interviews (KIIs) with members of the sub-County Health Management Team (SCHMT) and health workers who provide ECD services. This was to seek their opinion on how the messaging platform influenced caregivers’ practices, their relationship with their children and how the intervention benefited ECD- practices in the community.

The interviews were conducted through eight focus group discussions with 99 participants including primary caregivers (n = 82) and community health volunteers (CHVs)(n = 17), IDIs with 5 caregivers and 4 CHVs who had received and supported the intervention respectiveily and key informant interviews interviews with health managers and workers (n = 3).

Information on the number of respondents in each interview type is presented in Table 1.

**Table 1:** Characteristics of the study participants

Focused group discussions	N	IDIs and KIIs	N
**Age (years)**			
< 20	5	< 20	1
20–35	94	20–25	6
		> 35	5
**Marital status**			
Never married	11	Married	9
Married	79	Single	1
Widowed	2	Widowed	2
Divorced	4		
Single	3		
**Education level**			
Kindergarten	1		
Primary	58	Primary	7
Secondary	38	Secondary	3
Tertiary	2	Tertiary	2
**Occupation**			
Unemployed	38	Unemployed	4
Employed	26	Employed	7
Self employed	38	Self employed	1
Total	99	Total	12

All interviews and discussions were facilitated by field interviewers who had a graduate-level qualification in Early Child Development and Education or social sciences with experience in conducting qualitative interviews. They were trained over five days by the research team on qualitative interviewing methods, research ethics and the interview questions through ” classroom” sessions, role plays and a field pilot. The piloting exercise tested and provided feedback on the interview guide and ensured that the field interviewers had a good understanding of it.

Every interview was conducted by two field interviewers, one moderating the discussion and the other taking notes. The FGDs and IDIs with caregivers were administered in Kiswahili, the commonly used language in this locality while KIIs were carried out in English. All interviews were audio recorded as they were on-going to mitigate data loss. FGDs were 60 min long while IDIs and KIIS took about 45 min. The quality of the interview process was enssured by a research officer from the research team who was debriefed by the interviewers, and reviewed the notes after each interview was completed. This provided a mechanism to assess if saturation was being achieved in the data being collected.

### Data analysis

The audio recordings were transcribed and translated into English by a qualified and experienced transcriber. The transcripts were checked for accuracy and quality assurance by the Research Officer (who is the first author) on the study team.

The transcripts were then manually coded using a coding scheme, prepared by the lead author and other researchers in the team, that was based on predetermined themes in line with the study aims and research questions [15]. Coding was done by five researchers working independently and then reviewed by two members of the team to establish consistency in the application of codes. The few discrepancies were resolved by the team. Other emerging themes were arrived at through identifying patterns, defined as concepts that occurred repeatedly across the different transcripts. Other underlying factors were coded as subthemes under related themes guided by the codebook.

### Ethical considerations

The study protocol and tools were reviewed and approved by the African Medical Research Foundation (AMREF) Ethics and Scientific Review Committee (REF No. P578/2018). Written informed consent was obtained from all the interview participants before conducting the interviews. The study was conducted in accordance with the principles of the Declaration of Helsinki and no incentives were offered for participation.

## Results

We present the results with a focus on caregiver knowledge, attitudes and practices. Interviews held after six months and twelve month of the intervention, showed changes in caregiver knowledge, attitude and practices, perspectives that were also observed by CHVs and health workers.

### Reported improvements in caregivers’ knowledge of ECD and caregiving

At midline, caregivers, CHVs and health workers mentioned that participating in the intervention was beneficial with regards to enhancing caregiver knowledge and understanding of ECD (developmental milestones) and caregiving and equipping them with new information. This is because information on the developmental progression of their children had been made available to the caregivers through the monthly text messages that they recieved. Further, caregivers mentioned that the suggestions that they had received on how to stimulate their children enhanced how they attended to and cared for their children.

*“This project has helped me a lot in the development of my child as there are things my child was not previously able to do. As soon as the question comes in, I look for the child and she practically does as per the question. If it is a question about a spoon, l give her food and observe if she can feed herself using that spoon then l respond to the question. I do not respond to the question out of my imagination on whether the child can or cannot perform the task. I do it practically so that l may respond to the question.”* FGD with female caregivers, Korogocho

Further, female caregivers reported that the program had provided them with a greater understanding of how children’s development takes place in stages and that they had become closer to their chidren and more attentive to their developmental changes. They reported that the messages that caregivers received also served as a reminder of the activities they could engage in together with their children as learning opportunities.

*“...It has really helped me because l have learnt that as a child grows, things develop one stage after another and she does not have to do all the things at one go. She will develop them one at a time as she continues to grow…”* FGD with female caregivers, Korogocho

*“…In my view the project has really helped me because those questions act as a reminder. Every time they ask me “…can your child do this?” I go and try out with her. If l observe she can do it…and then they tell me to show her how to do something, l show her and she learns to do it...”* FGD with female caregivers, Korogocho

*“...I have observed that there is a big change because l sometimes find him playing. He takes the ball and throws it to me; if l throw it back he enjoys the game. When we get to the house maybe he wants his book and starts to draw in it. He even starts telling me this is this and the other…”* FGD with male caregivers, Korogocho

There was information and knowledge sharing with other members in the caregivers’ households. Caregivers reported that they were able to inform/teach other members of their families on what they had learnt from the intervention with regards to how to handle or play with the child. Caregivers also reported that they shared this information with other parents in the community. This increased the awareness and potential for improved practices on child stimulation within and beyond the household.

...I would say, there is a time the father got a question and asked me if a child is supposed to open books and view pictures. I told him yes, that these questions are to assist us to know what to do for the child. The next day he went and bought books and crayons for the child. So in the evenings, he calls the child and they begin to color. So now the child already knows when his father comes in, they take the books and they begin to color…

FGD with female caregivers, Korogocho

Increased caregiving knowledge among the caregivers receiving the intervention was also observed and noted by the CHVs who supported the intervention. They, (CHVs) mentioned that caregivers (mothers and fathers) had become more aware of the importance of ECD monitoring. They further noted that caregivers had acquired knowledge on how to do it well particularly with regards to monitoring their children’s milestones and in supporting the child to achieve the expected milestones through stimulation.

Simillar sentiments were echoed by a health worker and CHVs who supported the interventions. In both instances they indicated that the mobile phone platform was beneficial in providing mothers with information and sensitizing them on the development of their children.

A health worker at the community health facility said that through the monthly messaging, caregivers were better placed to follow up on their children’s development and note anormalies.

…Before that, you find that the same caregivers will just stay, until maybe a CHV will come later when sometimes it is already too late to be helped to achieve those milestones. But with the phone, they are able to know to follow their children well and see if they are achieving these milestones. So for the children it is easier for these caregivers to help them, to achieve the milestones, so we have less children who are not achieving milestones, less children who are … malnourished, we have less people on the program right now because now the parents know the things they are supposed to look for, for the children...

KII with health worker, Korogocho

…when the caregivers started being asked questions they begun to realize that it is important because the child begins to understand things early or to understand things well…so the caregiver begins to appreciate that they should teach the child things like these and the children begin to learn things that they do not know. Even playing ball with a child in that age bracket that we have had in the Saving Brains project, a caregiver does not know that they can take up a ball and kick it with the child…is important in the growth of a child. There are parents like those and we have them in the community and they are now becoming knowledgeable…

IDI with CHV, Korogocho

### Reported changes in attitudes towards ECD and caregiving

We noted that many caregivers pointed out that they appreciated their children better and felt more capable of responding to their developmental needs more appropriately than they did before they were exposed to the intervention. One caregiver said that the intervention demonstrated that caring for a child was not that difficult and participating in the project had given them confidence in their caregiving as indicated in the quotes below.

For me, l now have confidence that l can care for my child. As the questions keep coming l keep practicing those things with my child. Before l did not believe that l could play with my child or be like a parent. But now l have confidence…

FGD with female caregivers, Korogocho

Female caregivers and CHVs reported that the intervention had encouraged increased male participation in the care of young children as they (male caregivers) received information on how to care for and relate with their children. In some cases, female caregivers did not own a phone and resulted to sharing a phone with their male spouses. This occurrence gave male spouses an opportunity to engage with the intervention partly or jointly and therefore receive nurturing information like never before.

*“...For me it has really helped because the father of this child never wanted to see the child, even when the child wanted to play with him, he did not want. But now l observe that he will even pick the toys, they sit together and begin to play…”* FGD with female caregivers, Korogocho

CHVs also noted that as a result of sharing phones in the case where a household depended one phone to receive and send messages, fathers had become interested and more involved in understanding their children’s development and implementing what was advised for the child to achieve the milestones.

*For me, my child and his father are not usually very close as it is difficult for my husband to be around daily or he returns when the child is already asleep. But when he is around, they play together and he checks on the messages to see what the child can do even though l am the one who responds…*

FGD with female caregivers, Korogocho

We (CHVs) are really happy because even the men have come to understand about their children because they receive the questions and they too are happy to respond. It is making them happy because they can see the children are progressing step by step getting to know that a child needs to breastfeed, a child needs to play so they are happy.

FGD with CHVs, Korogocho

Male caregivers reported that participating in the project helped them know that the role of stimulating development and learning in young children was not the preserve of a female caregiver. They now understood that children play as part of their learning and play was achievable and not a difficult thing to do with a young child.

...Every so often we men we take it as though caring for young children is the burden of the women but this project has enlightened us and gotten us to know that we need to do it together to help with caring for the child…

FGD with male caregivers, Korogocho

...This project has helped because previously before it brought us this education, we knew that playing with a child is wrong. That if you put a ball to kick and the child also kicks, that is wrong…So now if l give the child a pencil, they may spoil it while wanting to know what is inside that is making it draw on this book and there is nothing wrong with it. You leave the child to explore… many times most people in the slum understood that only the mother can play with the child things like those. So we (now) know that one can play with the child when you want to teach them something…

FGD with male caregivers, Korogocho

### Reported change in caregiving practices

Caregivers reported that as a result of participating in the intervention, they saw the benefit of being close to the child and spending time with the child. They reported that they had learnt the importance of play and were implementing this with their children. They also began seeing and utilizing learning opportunities with their children. This sentiment was expressed by both female and male caregivers who participated in the intervention. A few caregivers reported that their use of harsh discipline (disciplining a child by inflicting some pain) had reduced in that they no longer saw it as the best way to communicate to a child that they had done something wrong.

…For me, it has helped me to be close to both my children. It has also helped me understand that with a routine, a child gets used to doing things like l can give her a cloth item for her to dress herself. Before, if l would give her a cloth item and she wears it wrong, I would beat her. Now l know l need to teach her so that she can know. I have also learnt that you can give a child a book to read, so now she can count even though not consistently like …1, 2, 5… I have learnt that l can teach her…

FGD with female caregivers, Korogocho

…l used to think that playing with a child was bad. When they (assessors) came home and l saw how when they put things there like stones to pile them, the child watched this and then also imitated and when they fall the child would laugh. Thereafter they said l should show the child how to play. After that l also started making the child play. I bought some things, l cut up some wood pieces and when l place three or four or five, when they fall she laughs, then she places more. That is how a child should play. So as she grows, she knows it is a good game…Now she cannot play a bad game when l am close-by…

FGD with male caregivers, Korogocho

*“...It has taught me that the cane does not help. Previously, l was one to cane the child l used to think that the cane is what teaches the child. I have also become friendly with the child. Yes…When she spoils something, l have also learnt that she would have curiosity and I would interpret that as her wanting to spoil something so l would beat her with a cane. But now l have learnt that she is curious and wants to know and l have learnt how to be friendly…”* FGD with female caregivers, Korogocho

These changes in caregiving were also observed by the CHVs who mentioned that caregivers had become keen on monitoring their children’s development.

…What has been most successful as l see it is that the children have improved in the slum. That is why as l had mentioned earlier, someone leaves the child in the child daycare center and is not bothered on how the child is developing as long as they look well and they are breathing and there is no sign of illness anywhere. When they get to the house, tomorrow they go back. However now at least now there is a parent whom you could ask how the child is progressing and that is the improvement you will see in the slum. At least when you ask the mother, even before you ask her she will tell you my child is progressing in this or this way”. Therefore, l see it is improving...

FGD with CHVs, Korogocho

A community health assistant expressed hope, after noting the change in caregiving behavior. She mentioned the cascading effect that good caregiving would potentially have in future for the caregivers. That this trend would provide for less worried parents who could engage with economic activities and provide better for their children, not held back by children who are not thriving.

*“If the children are okay, that means these parents don’t have to take their children to the facilities now and again, that saves time and that saves money. If the babies are well developed, it means the parents don’t have to worry about these kids so they can be able to go out there and…be able to do something to get some income…They will be at least economically stable. They will not use a lot of money on medical care or rather making sure these kids are well. It also gives again the parents peace of mind which is the most important.”* KII with CHA, Korogocho

From the data collected we see that caregivers obtained knowledge and were encouraged to have a means through which to know how their children are growing. They appreciated that there is something they could do contribute towards their children’s development. We see caregivers sharing these learnings out of their own volution with other family and community members. The knowledge acquired through the SMS platform had made a difference in their caregiving practices. We also see that in some cases, this experience was shared between male and female caregivers in a home.

## Discussion

This study set out to explore the influence of a mobile phone technology-based ECD intervention on the caregivers’ ECD knowledge, attitudes and practices. From the evidence provided, there is a gap in knowledge and practice among caregivers living in informal settings. The responses from caregivers indicate that there was acquisition of new knowledge on milestones and child development that made a difference. Caregivers gained insights on what to observe and how to make observations on their children to acertain proper development. Caregivers’ responsiveness to changes in the development of their children was enhanced through the activity cues that they received via the sms platform. The messages they received triggered active engagement and assessment of their children consistently. The platform additionally provided an opportunity for male caregivers to learn how they could engage with their young children. This was a departure from the evidence at baseline where caregiver knowledge on early child developmental changes was limited, as was involvement in play and stimulative activities with their young children [5]. Low ECD knowledge and poor childcare practices is not a unique problem in impoverished settings. Poverty and low education levels have been implicated in poor childcare knowledge and practices in these settings [16–18]. Caregivers rely on limited information given during counselling services when they attend child services at the health facility and comparision of their children to other proximal children. The information received in clinical setting is usually devoid of information child development and stimulation details as narrated in the baseline interviews [5]. Our evidence shows that this intervention filled this knowledge gap and improved caregiver understanding of their childrens’ staged development while boosting their ability and confidence to respond to it. This study demonstrates how a phone based intervention can resolve limitations of a health care system in providing information and knowledge to meet the caring needs of children.

The evidence in this study depicts instances of changes in caregiving attitude. Caregivers better appreciated their role in playing with their children and the relevance it has in the development of their children. Caregivers begun to appreciate items in their homes that children could play with. The sms-platform affected gender attitudes in caregiving. We see evidence of male caregivers becoming motivated to connect with their young children as they understood that play is developmentally supportive for their children. Cultural and religious practices that undermine the uptake of healthly caregiving practices in the African context are common, even more in low-income settings as has been the case with the population in this study [5]. Overall, the sms platform motivated caregivers to change their care practices with their children. They reported taking note of their childrens cues for play and intentionally engaging in play with their children. Also, there was change in how they noted the need to assist their young children to develop age-appropriate skills around the home and not dismis such opportunities as before.

This study provides evidence that a customized digital solution can change child care practices and behavior norms by intergrating currently deficient, yet necessary knowledge that motivates responsive care by both female and male caregivers. We attribute this to the simplicity, relatability and the convenience of that the mobile platform provided for use by caregivers in living in informal settlements. It promoted motivation and self-efficacy among the caregivers which is noted as crucial in the success of mobile based interventions [19]. Because employment is noted as a key factor in the involvement of male caregivers in active caregiving [20], this mobile-based instrument shows the potential to increase much needed knowledge to male caregivers on how to interract with their young children conveniently and in a context where lack of knowledge explains the caregiving norms [21].

We note that the information sent to the caregivers in the intervention was shared with other household and community members. We view this as a positive milestone that suggests the sustained influence potential of the intervention. That caregivers in the community become catalysts of knowledge and attitude change for care practices of young children. Evidence from other settings [22] shows that when community members support and engage with one another, this could provide peer-coaching around good caregiving that also functions to support memory and retention of information being provided while changing attitudes toward caring for children. This observation also suggests that the potential positive change in caregiving and child ECD outcomes is not only limited to the children participating in the intervention but also children born in the future to beneficiaries of this knowledge.

### Limitations

The intervention run for twelve months and despite the relatively short duration, it was perceived to improve the knowledge and practices of caregivers. It is possible that a longer implementation period could have resulted in much stronger changes and allow for time to make clear behavior observations in caregiver-child interractions.

## Conclusion

This study establishes great potential in utilizing mobile phone technology in enhancing caregiver knowledge, attitudes and practices. The study shows that ditigal instruments surpass a variety of barriers to meeting needs in caregiving in the early years. Wheather as an instrument of knowledge provision, informing practice or changing behavior and attitudes, a good grounding for such a platform is in it’s co-designing with potential users to effect motivation for its use and sustainability. It is important to investigate how a similar intervention intended for male caregivers would inform and influence care practices for them and address attitude and behavior changes towards responsive caregiving. Other aspects of caregiving in the early years including child feeding, child wellness and health could also be incorporated as per the need of the population tartgetted. As such, we recommend further work in these areas.

### Electronic supplementary material

Below is the link to the electronic supplementary material.

**Supplementary Material 1**

## Data Availability

The datasets used and analysed during the current study are available forom the corresponding author on reasonable request.

## Declarations

We briefed each participant on the study and its objective after which we obtained written consent from each participant to participate in the study and interviews. This process was undertaken using consent forms to provide details on the study including the objectives, the procedures, their rights with regrads to voluntary participation, potential risks and benefits, and obtain their approval. We also issued a copy of this consent to each of the study participant to keep for themselves.

## Abbreviations

CHV: Community Health volunteer
ECD: Early child development
